# Supplementary material for: Evolution to alternative levels of stable diversity leaves areas of niche space unexplored
Source: PLoS Comput Biol. 2021 Jul 28;17(7):e1008650. doi: 10.1371/journal.pcbi.1008650 (PMC8351994; doi:10.1371/journal.pcbi.1008650)
Supplement: S1 Supporting Information — Full model details. Full description of equations, simulation algorithm, and modeling methodology. Numerical stability analysis. Numerical stability analysis was performed on each simulation referenced in the text in order to confirm meta-stability. PDE model. Evolutionary dynamics when model is formulated as a partial differential equation model. (PDF) [file pcbi.1008650.s001.pdf]

Evolution to alternative levels of stable diversity leaves areas of niche space unexplored

Ilan N. Rubin[1*], Iaroslav Ispolatov[2], Michael Doebeli[1,3]

1 Department of Zoology, University of British Columbia, Vancouver, British Columbia, Canada
2 Departamento de Física, Universidad de Santiago de Chile (USACH), Santiago, Chile
3 Department of Mathematics, University of British Columbia, Vancouver, British Columbia, Canada

* rubin@zoology.ubc.ca

S1 Supporting information

Full Model Details.

We consider a general model of logistic growth and frequency-dependent competition based on a d-dimensional phenotype [1, 2]. For simplicity the model does not consider spatial interactions. Evolution is modeled in three ways: adaptive dynamics, an individual-based model, and partial differential equations.

Ecological Dynamics

Ecological dynamics follow logistic growth governed by a carrying capacity $K(\vec{x})$ and a competition function $\alpha(\vec{x}, \vec{y})$ where $\vec{x}$ and $\vec{y}$ are the phenotypes of competing types with $\vec{x}, \vec{y} \in \mathbb{R}^d$.

The competition function is defined such that $\alpha(\vec{x}, \vec{x}) = 1$, and for symmetric competition $\alpha(\vec{x}, \vec{y}) < 1$ for $\vec{x} \neq \vec{y}$. Thus, the Gaussian part of competition between types diminishes as their phenotypes becomes more distant and is maximal between individuals with the same phenotype. For symmetric competition the competition function is strictly Gaussian.

$$\alpha(\vec{x}, \vec{y}) = \exp\left(-\sum_{k=1}^{d} \frac{(x_k - y_k)^2}{2\sigma_\alpha^2}\right) \tag{1}$$

For asymmetric competition, the competition function also includes an additional term.

$$\alpha(\vec{x}, \vec{y}) = \exp\left(\sum_{k,l=1}^{d} b_{kl}(x_k - y_l)x_l - \sum_{k=1}^{d} \frac{(x_k - y_k)^2}{2\sigma_\alpha^2}\right) \tag{2}$$

This first term is the first order of Taylor expansion of a higher order, non-symmetric competition function and thus represents the simplest form of adding non-symmetric dynamics to the Gaussian function [1]. It includes the coefficients b_{kl}. For the non-symmetric competition simulations discussed here, a specific set of b coefficients were chosen that resulted in periodic evolutionary dynamics [provided in **??**]. The models were also run with many other values for these coefficients, including a survey of randomly chosen values, but results were qualitatively similar to the values chosen. For

a review of how these values govern evolutionary dynamics in higher dimensions, please see Doebeli et al. [2].

The carrying capacity function $K(\vec{x})$ represents the equilibrium population size of a population of only individuals with phenotype $\vec{x}$. Two carrying capacity functions are discussed here. What is the quartic carrying capacity is defined as such.

$$K(\vec{x}) = \exp\left(-\sum_{k=1}^{d} \frac{\vec{x}_k^4}{4}\right) \tag{3}$$

The radially symmetric carrying capacity is as follows.

$$K(\vec{x}) = \exp\left(-\frac{\left(\sum_{k=1}^{d} \frac{\vec{x}_k^2}{2}\right)^2}{2}\right) \tag{4}$$

Both functions are similar in that they are maximal at the origin and of the fourth order. This ensures that there is stabilizing selection toward $\vec{x} = 0$ and that the phenotype space that is viable is bounded near the origin. The quartic carrying capacity function has an approximately square peak, while the radially symmetric function is circular [Fig. 1].

Together, ecological dynamics are as follows,

$$\frac{dN_i}{dt} = rN_i(t)\left(1 - \frac{\sum_j^M \alpha(\vec{x}_j, \vec{x}_i)N_j(t)}{K(\vec{x}_i)}\right) \tag{5}$$

with N_i representing the population size of individuals with phenotype $\vec{x}_i$, in a total population of M different phenotypes, and an intrinsic growth rate of r.

Adaptive Dynamics

Using adaptive dynamics [3–5], the evolution of a phenotype $\vec{x}$ can be described by a system of differential equations $d\vec{x}/dt$. This is derived from the invasion fitness $f(\vec{x}, \vec{y})$, which is the per capita growth rate of a rare mutant with phenotype $\vec{y}$ in a resident population with phenotype $\vec{x}$.

$$f(\vec{x}, \vec{y}) = 1 - \frac{\alpha(\vec{x}, \vec{y})K(\vec{x})}{K(\vec{y})} \tag{6}$$

This invasion fitness function relates how the growth rate of the resident is always decreased with the introduction of a new mutant unless $\vec{x} = \vec{y}$ when $f(\vec{x}, \vec{y}) = 0$. From this we can derive the selection gradient, s, as the derivative of the invasion fitness with respect to the mutant, $\vec{y}$, when it is equal to the resident, $\vec{x}$.

$$s_i(\vec{x}) = -\left.\frac{\partial \alpha(\vec{x}, \vec{y})}{\partial y_i}\right|_{y=x} + \frac{\partial K(\vec{x})}{\partial x_i}\frac{1}{K(\vec{x})} \tag{7}$$

The adaptive dynamics are defined as

$$\frac{d\vec{x}}{dt} = \hat{M}(\vec{x})\vec{s}(\vec{x}) \tag{8}$$

where $\vec{M}$ is the mutation-covariance matrix describing the rate, size, and covariance of mutations in each phenotypic dimension. For simplicity we assume this is the identity matrix. Any formulation of this matrix with a positive diagonal would only change the

speed of the evolution in the different dimensions, but would not change the characteristics of any evolutionary dynamics or stable states. The adaptive dynamics are therefore a set of differential equations that describe the evolutionary dynamics in d dimensions for a given set of phenotypes.

Of note, while the Gaussian part of the competition kernel α affects whether diversification can occur and multi-species dynamics, because the selection gradient is evaluated at $\vec{y} = \vec{x}$, in the adaptive dynamics of monomorphic populations the Gaussian part of α disappears and we are left with just the effects of asymmetric competition. Because of the exponential nature of carrying capacity functions, the second term of the selections gradient, $\frac{\partial K(\vec{x})}{\partial x_i}\frac{1}{K(\vec{x})}$, reduces to just the partial derivative of the inner function with respect to the resident. For the quartic case the adaptive dynamics thus as follows

$$\frac{dx_k}{dt} = \sum_{l=1}^{d} b_{kl}x_l - x_k^3 \tag{9}$$

For the radially symmetric case the adaptive dynamics are

$$\frac{dx_k}{dt} = \sum_{l=1}^{d} \left(b_{kl}x_l - \frac{x_l^2}{2}x_k \right) \tag{10}$$

In order to include extinction and speciation events, an iterative algorithm is used: (1) solve for the ecological dynamics; (2) remove any newly extinct populations that fall below a minimum viable population size; (3) solve the adaptive dynamics for a given length of evolutionary time; (4) introduce a new mutant, with a phenotype a small, fixed distance from one of the resident populations or with a phenotype chosen from a Gaussian distribution with mean equal to the phenotype as one of the resident populations and a given variance σ^2_{mut}; (5) remove the mutant if it is not ecologically viable (invasion fitness of the mutant is negative); and (6) repeat the process until either an evolutionary stable state or a given amount of time is reached.

At given intervals, clusters are calculated using a hierarchical clustering algorithm such that any two population with phenotypes within a small distance, z_{small}, from each other are part of one cluster. Clusters are just an accounting device and do not affect dynamics. All parameters used can be found in **??**.

Individual-Based Model

The individual-based model uses the same ecological dynamics as described above and simulated based on the Gillespie algorithm [6]. Individual birth rates are assumed to be constant and equal to 1, while death rates, δ, are frequency dependent and derived from the ecological dynamics.

$$\delta_i = \sum_{j \neq i} \frac{\alpha(\vec{x}_j, \vec{x}_i)}{K(\vec{x}_i)K_{max}} \tag{11}$$

Here the competition function, α, and the carrying capacity function, K, are the same as in the adaptive dynamics, while K_{max} is a constant that controls the height of the carrying capacity function to convert it from continuous to discrete populations. These rates are taken directly from the Lotka-Volterra equations, where the per capita growth rate of $\left(1 - \frac{\sum_j^M \alpha(\vec{z}_j,\vec{z}_i)N_j(t)}{K(\vec{z}_i)}\right)$ can be considered the birth rate minus the death rate. The individual-based model is thus a direct analog of the adaptive dynamics. For a larger discussion on the derivation of the individual-based dynamics of this model please refer to [7–9].

The simulation algorithm is as follows: (1) initiate the population with a randomly chosen initial population of a predetermined size; (2) update or calculate all individual

death rates; (3) calculate the sum of all birth and death rates, $U = \sum_i (1 + \delta_i)$; (4) increment time by a random amount drawn from an exponential distribution with mean equal to $1/U$; (5) chose a random birth or death with probability equal to the ratio of the rate and the sum of all rates; (6) if a death rate is chosen, remove the chosen individual; if a birth rate is chosen, create a new individual with phenotype chosen from a Gaussian distribution with mean equal to the parent phenotype and variance σ^2_{mut}; (7) rerun steps 2-6 until the population is extinct or a specified time is reached.

Phenotypic clusters are calculated using a hierarchical clustering algorithm in which every individual in a cluster has a phenotype within a small distance of at least one other individual in the cluster. Individuals are added to a cluster at birth or a new cluster is created if the individual does not fit in any existing cluster. A cluster is updated when a member individual dies to see if the cluster splits. All parameters used can be found in **??**.

Numerical stability analysis.

While we were unable to analytically determine the stability of the communities that arose in our simulations, we were able to test for evolutionary metastability numerically. However, this method is computationally expensive, so we were only able to test for stability on a small number of representative simulations.

To test the stability the final population in a simulation we extensively sampled random mutants around every resident in the community (250 mutants per resident). For each new mutant, we continued the adaptive dynamics simulation for a significant period of time (5% the length of the original simulation), while not allowing any other branching mutations to arise. We then checked whether the original residents and the mutant were all able to survive ($N_i > N_{small}$) and the mutant was able to diversify ($|\vec{z}_m - \vec{z}_i| > z_{small} \forall i$ for a mutant with phenotype z_m and residents z_i) from the resident population. Any simulation in which no mutant can invade, coexist, and diversify is deemed an evolutionarily stable community.

While this method is unable to definitively prove stability as it is always possible that an extremely rare mutant could diversify or drive multiple residents extinct, we can at least demonstrate the metastability (as defined in the main text; any state that is maintained for a period significantly longer than its convergence) of these communities. An illustration of metastability can be seen in Figure A. Here, the convergence to the metastable cycle with 10 species takes approximately 25 evolutionary time units, after which the system resides in the metastable state until the end of the simulation, which lasts for another 975 time units. A clearer depiction of the early convergent dynamics can be seen in the main text in Figure 3.

This numerical stability analysis was used to test each simulation illustrated in the main text (Figs 2 and 4). Using the same small mutations as the original simulations, not a single mutant (of over 30,000 tested) was able to invade, coexist with, and differentiate from the original community. The same was true when we instead tested Gaussian mutations ($\sigma_{mut} = 0.05$). Only when we tested larger mutations (Gaussian mutations with $\sigma_{mut} = 0.1$) were 7 mutants (0.02% of those attempted) from two different simulations able to break the stability and diversify the community into a new, higher diversity state.

At least in the representative simulations tested, only large or extremely rare mutants are able to shift the community, proving that these states are indeed evolutionarily metastable.

Figures from the numerical stability analysis for each of these simulations can be found on-line at `https://www.zoology.ubc.ca/~rubin/AltEvoDiversity/`.

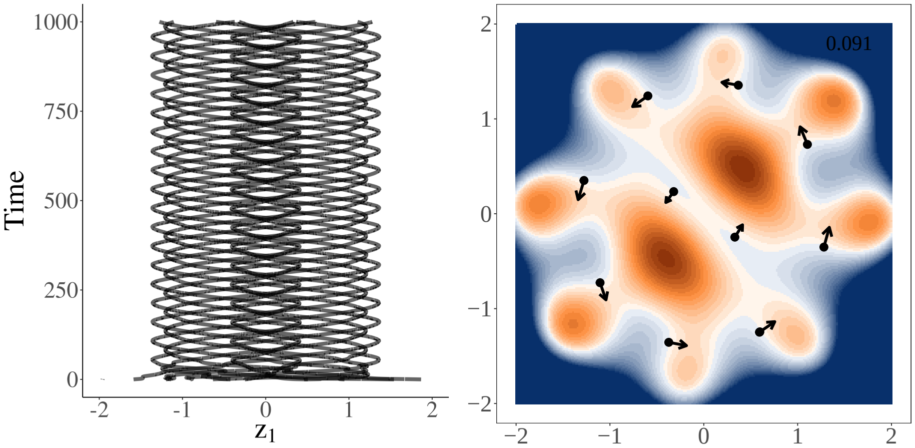

Fig A. Convergence to metastable limit cycle. Here we define metastability operationally as any system that resides in a state much longer than it took to converge to it. Here, the system converges to a stable limit cycle in approximately 25 time steps and remains there for the duration of the simulation. The simulation were run using the radially symmetric carrying capacity and initiated with 18 random species. Panel A shows the complete history of evolutionary dynamics. Panel B is a depiction of the population at the end of the simulation. Colors in panel B represent the invasion fitness (per capita growth rate of a new mutant if it were to arise). Positive invasion fitness is shown in shades of orange (maximum of 0.091), negative in blue, and invasion fitness equal to zero in white. Arrows are proportional to the square root of the selection gradient for each species.

PDE model.

In addition to the adaptive dynamics and individual-based models, we also ran partial differential equation simulations. PDEs represent the infinite population approximation of individual-based models [7, 9] and are useful for relaxing the phenotypes represented as a delta function and the separation of timescale between ecology and evolution assumptions of adaptive dynamics without the stochasticity of individual-based models.

The infinite population version of the individual based model gives the deterministic formulation of partial differential equations.

$$\frac{\partial N(\vec{x},t)}{\partial t} = N(\vec{x},t)\left(\frac{1-\int \alpha(\vec{x},\vec{y})N(\vec{y},t)d\vec{y}}{K(\vec{x})}\right) + \delta_{mut}\sum_{k=1}^{d}\frac{\partial^2 N(\vec{x},t)}{\partial x_k^2} \tag{12}$$

Here, $N(\vec{x},t)$ is distribution of the population with phenotype $\vec{x}$ at time t and δ_{mut} is diffusion coefficient that can be thought of as analogous to the rate of mutations and will not have qualitative effect on our simulations. The competition and carrying capacity functions are the same as described above. The PDE is numerically solved over a lattice. Because of memory limitations, using a high resolution lattice is infeasible, though we were able to run all 2-dimensional PDE simulations on a 200 x 200 lattice. This lattice is more than detailed enough to reveal any peaks and patterns that appear. The local maxima of the resulting distributions represent centers of clusters of individuals in the individual-based model.

Notably, like the individual-based model, the PDEs are not restricted to approximation of adaptive dynamics that phenotypes are represented as delta functions.

Instead, the distributions produced by the PDEs "take up space" in the phenotype space. This can result in configurations with fewer local maxima than adaptive dynamics populations when run with the same parameters. Additionally, because the PDEs are a diffusion process, configurations cannot be trapped by small mutations. This makes the PDE simulations unsuited to studying the presence or absence of alternative stable configurations. They are however an useful comparison to the adaptive dynamics and individual-based models that are fully deterministic and free of the assumptions intrinsic to adaptive dynamics.

When PDE simulations are run with symmetric competition, the resulting population densities are configured in the same 4x4 grid and two concentric circles as the comparable adaptive dynamics simulations (Figs 2, B). The same concentric circles appear as ridges of high population density, but only the inner is peaked and far less distinctly than with the quartic grid. As the adaptive dynamics assumes species can be represented by a single phenotype without variance, continuous diversification around the circles is the only way for the population to mimic the circular ridge predicted by the PDE. This implies that with infinite population size (adaptive dynamics and PDE), simulations with symmetric competition and a radially symmetric carrying capacity eventually (though this process is slow) result in no distinct species or phenotypic clusters. However, as noted in the main text, this situation of continual diversification along concentric circles in trait space is likely a degenerate case that is cause by a radially symmetric carrying capacity function and Gaussian competition.

Within species phenotypic variation reduces diversity

For the asymmetric competition, PDE simulations with both quartic and radially symmetric carrying capacities result in similar, but less diverse patterns in comparison to the highest evolutionary stable diversity state from the adaptive dynamics simulations (10 versus 14 phenotypes for the quartic carrying capacity and 14 versus 16 phenotypes for the radially symmetric case) (Figs 4, B). The reduced diversity is likely because the peaks in the adaptive dynamics are delta peaks, while those in the PDE have non-zero variance in phenotype space, restricting the number of distinct peaks. The radially symmetric carrying capacity simulation did exhibit the same clockwise rotation in both rings as displayed in the adaptive dynamics and individual-based models (video available in on-line, **??**).

There has been previous theoretical work that predicts variation within species clusters has a negative effect on coexistence between competing species [10]. As species now occupy a distribution in trait space, rather than a single point, niche differentiation between species is reduced, impeding the maintenance of higher numbers of distinct phenotypes. As the PDE is the infinite population limit of the individual-based models, we would expect the same lower diversity at the global ESC when the individual-based simulations are run with very large communities and mutation sizes greater than some ϵ (these simulations were computationally infeasible to run). While we don't expect the other results (the presence of locally stable ESSs or limit cycles) from this paper to be affected by the lower diversity, we feel it is a salient point worth considering when comparing theoretical models of diversification to expectations for natural populations.

All parameters used can be found in **??**.

References

1. Doebeli M, Ispolatov I. Chaos and unpredictability in evolution. Evolution. 2014;68(5):1365–1373. doi:10.1111/evo.12354.

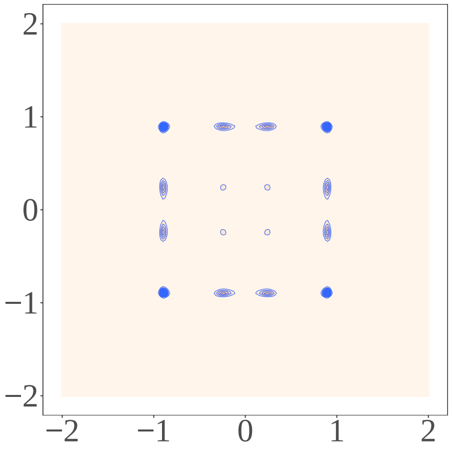

(a) Symmetric competition; Quartic carrying capacity

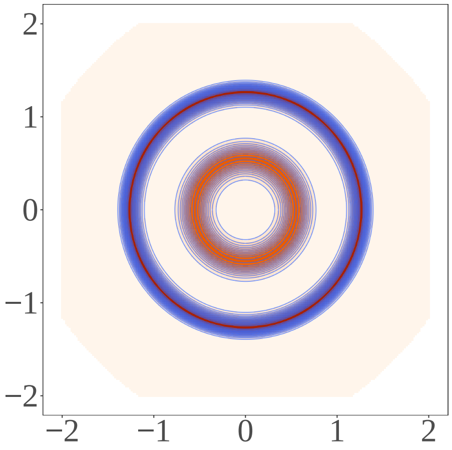

(b) Symmetric competition; Radially symmetric carrying capacity

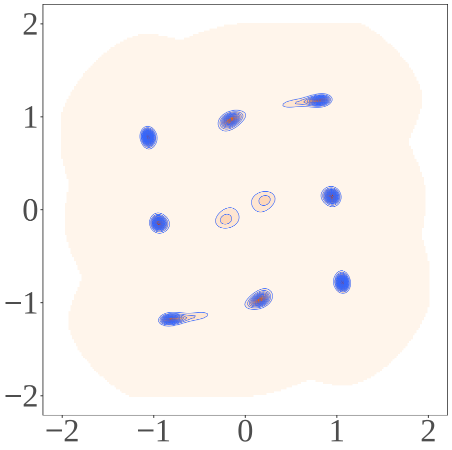

(c) Asymmetric competition; Quartic carrying capacity

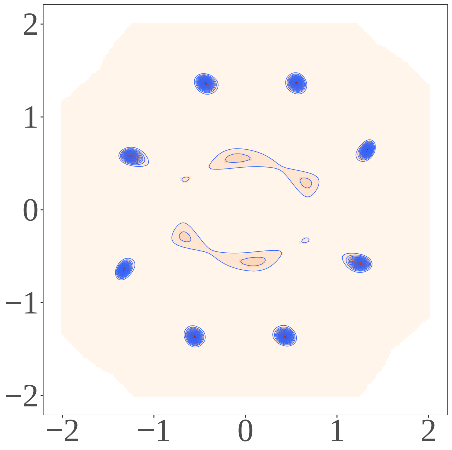

(d) Asymmetric competition; Radially symmetric carrying capacity

Fig B. The final population density when simulated using a PDE. Higher densities are darker and lower densities are lighter. Contour lines are shown in blue. 3-dimensional, interactive versions of the same figures and videos of the dynamics are available on-line (**??**). Population distributions generated using the PDE models result in similar configurations to those emergent from the Adaptive Dynamics, but with fewer distinct species resulting from intra-species phenotypic variance. The asymmetric competition and radially symmetric carrying capacity simulation exhibits the same clockwise rotation displayed in the adaptive dynamics and individual-based simulations.

2. Doebeli M, Ispolatov Y. Diversity and Coevolutionary Dynamics in High-Dimensional Phenotype Spaces. The American Naturalist. 2017;189(2):105–120. doi:10.1086/689891.

3. Metz JAJ, Nisbet RM, Geritz SAH. How should we define ‘fitness’ for general ecological scenarios? Trends in Ecology & Evolution. 1992;7(6):198–202. doi:10.1016/0169-5347(92)90073-K.

4. Dieckmann U, Law R. The dynamical theory of coevolution: a derivation from stochastic ecological processes. Journal of mathematical biology. 1996;34(5):579–612. doi:10.1007/BF02409751.

5. Doebeli M. Adaptive Diversification (MPB-48). Princeton University Press; 2011.

6. Gillespie DT. A general method for numerically simulating the stochastic time evolution of coupled chemical reactions. Journal of Computational Physics. 1976;22(4):403–434. doi:10.1016/0021-9991(76)90041-3.

7. Ispolatov I, Madhok V, Doebeli M. Individual-based models for adaptive diversification in high-dimensional phenotype spaces. Journal of Theoretical Biology. 2016;390:97–105.

8. Dieckmann U, Doebeli M. On the origin of species by sympatric speciation. Nature. 1999;400(6742):354–357. doi:10.1038/22521.

9. Champagnat N, Ferrière R, Méléard S. Unifying evolutionary dynamics: From individual stochastic processes to macroscopic models. Theoretical Population Biology. 2006;69(3):297–321. doi:10.1016/j.tpb.2005.10.004.

10. Hart SP, Schreiber SJ, Levine JM. How variation between individuals affects species coexistence. Ecology Letters. 2016;19(8):825–838. doi:10.1111/ele.12618.
