# Supplementary material for: Evolution to alternative levels of stable diversity leaves areas of niche space unexplored
Source: PLoS Comput Biol. 2021 Jul 28;17(7):e1008650. doi: 10.1371/journal.pcbi.1008650 (PMC8351994; doi:10.1371/journal.pcbi.1008650)
Supplement: S1 Text — Additional supplemental files can be found at https://www.zoology.ubc.ca/~rubin/AltEvoDiversity/. (PDF) [file pcbi.1008650.s002.pdf]

Evolution to alternative levels of stable diversity leaves areas of niche space unexplored

Ilan N. Rubin[1*], Iaroslav Ispolatov[2], Michael Doebeli[1,3]

1 Department of Zoology, University of British Columbia, Vancouver, British Columbia, Canada
2 Departamento de Física, Universidad de Santiago de Chile (USACH), Santiago, Chile
3 Department of Mathematics, University of British Columbia, Vancouver, British Columbia, Canada

* rubin@zoology.ubc.ca

S1 Text.

Videos, additional figures, and model source code.

Videos of the evolutionary dynamics for all simulations highlighted in the paper (including adaptive dynamics, individual-based, partial differential equation, and stability analysis simulations) can be found on-line at `https://www.zoology.ubc.ca/~rubin/AltEvoDiversity/`. Additional figures including 3-dimensional, interactive landscapes of the final population density of PDE simulations can be found here as well. Source code for the model is also included.
