# Supplementary material for: Evolution to alternative levels of stable diversity leaves areas of niche space unexplored
Source: PLoS Comput Biol. 2021 Jul 28;17(7):e1008650. doi: 10.1371/journal.pcbi.1008650 (PMC8351994; doi:10.1371/journal.pcbi.1008650)
Supplement: S1 Parameters — Table A. Default parameters for adaptive dynamics simulations. Table B. Default parameters for individual-based simulations. Table C. Default parameters for partial differential equation based simulations. Table D. Default asymmetric competition parameters. (PDF) [file pcbi.1008650.s003.pdf]

Evolution to alternative levels of stable diversity leaves areas of niche space unexplored

Ilan N. Rubin[1*], Iaroslav Ispolatov[2], Michael Doebeli[1,3]

1 Department of Zoology, University of British Columbia, Vancouver, British Columbia, Canada
2 Departamento de Física, Universidad de Santiago de Chile (USACH), Santiago, Chile
3 Department of Mathematics, University of British Columbia, Vancouver, British Columbia, Canada

* rubin@zoology.ubc.ca

S1 Parameters.

Default parameters for adaptive dynamics simulations.

Parameter	Value	Definition
σ_α	0.5	Standard deviation of the competition kernel
r	1.1	The intrinsic growth rate
ϵ_{mut}	0.005	Mutation size
T_{max}	10^5	Number of time steps simulations run for
T_{split}	0.2	Time between each branching attempt
v_{min}	10^{-10}	Evolutionary velocity* deemed stable to stop simulation early
N_{small}	10^{-8}	Population density which populations are considered extinct
z_{small}	10^{-3}	Phenotypic distance two species are deemed identical and merged

Table A. Parameters and variables used to generate adaptive dynamics data and figures.

*Evolutionary velocity is defined as the population weighted average magnitude of the rates of the phenotype vectors:

$$v = \sum_{i=1}^{M} \frac{N_i \sum_{k=1}^{d} |dx_{ik}/dt|}{\sum_{j=1}^{M} N_j} \tag{1}$$

After each step solving the adaptive dynamics (step 3 as outlined above), the evolutionary velocity is checked, and if $v < v_{min}$, the simulation is deemed stable and stopped.

Default parameters for individual-based simulations.

Parameter	Value	Definition
σ_α	0.5	Standard deviation of the competition kernel
r	1.1	The intrinsic growth rate
σ_{mut}	0.005	Standard deviation of mutation
T_{max}	10^5	Number of time steps simulations run for
K_{max}	400	The height of the carrying capacity function at the origin
$\delta_{cluster}$	$50\sigma_{mut}$	The maximum distance between individuals of the same cluster

Table B. Parameters and variables used to generate individual-based model data and figures.

Default parameters for partial differential equation based simulations.

Parameter	Value	Definition
σ_α	0.5	Standard deviation of the competition kernel
N_{bins}	200	The number of bins in each dimension
z_{range}	$(-2, 2)$	The minimum and maximum phenotype in each dimension
δ_{mut}	0.01	the amplitude of diffusion modeling mutation
T_{max}	10^3	Number of time steps simulations run for
σ_N	0.1	Standard deviation of the initial spread around the origin
δ_t	2	The integration time step

Table C. Parameters and variables used to generate partial differential equation model data and figures.

Default asymmetric competition parameters.

$$\begin{bmatrix} 0.40716468083977814 & -0.60799602828722576 \\ 0.94740291877661453 & 0.65155997501420926 \end{bmatrix}$$

Table D. The default values of parameter b that dictates the competition asymmetry used in all asymmetric competition simulations.
