# Supplementary material for: Evolution to alternative levels of stable diversity leaves areas of niche space unexplored
Source: PLoS Comput Biol. 2021 Jul 28;17(7):e1008650. doi: 10.1371/journal.pcbi.1008650 (PMC8351994; doi:10.1371/journal.pcbi.1008650)
Supplement: Attachment — Submitted filename: response_to_reviewers.pdf [file pcbi.1008650.s004.pdf]

Response to Reviewer Comments

Reviewer #1

(1) A core concept in this paper is the notion of local evolutionary stability – i.e., (meta)stability against small evolutionary perturbations. I would distinguish this from the somewhat related concept of stationarity – the tendency of a system to stay in a given state (or limit cycle) for some long period of time. Metastability and stationarity often go hand in hand, but the latter does not necessarily imply the former, particularly as the dimensionality of the dynamical system increases (as in this study).
My biggest concern is that many of the conclusions in this study are phrased in terms of stability, but much of the evidence is presented in terms of stationarity (e.g. reporting results after some long but finite simulation run). There are some exceptions – e.g., Fig. 1A convincingly demonstrates stability by plotting the relevant invasion fitnesses. But there are other places (e.g. lines 222-235) where even a demonstrated lack of stability seemed to be equated with a form of "de facto stability". Given the abstract nature of the model, it was not clear why slowness in simulations implies irrelevancy on "reasonable biological timescales" (line 231) without some additional effort to calibrate parameters. E.g. current estimates suggest that a single person's gut microbiota produces anywhere from 1e09 to 1e12 new mutations every day (Zhao & Lieberman et al, Cell Host & Microbe 2019), so 1̃e05 attempted branching events does not seem out of the question.
Together, these somewhat fuzzy notions of stability made it much more difficult for me to assess the central claims of the paper, particularly in cases where stable limit cycles started to dominate the dynamics. In these cases, the effective state space of the system (# distinct phenotypic arrangements x # of local mutations) would seem to grow quite large, making it difficult to exhaustively sample the local neighborhood as in Fig. 1A. How can we sure that true instabilities aren't getting swamped by the entropy of the local neighborhood?
My suggestion would be to try to remove some of the more speculative language about stability / the stability landscape (e.g. lines 403 & 417, or the use of "final evolutionary equilibrium" in Figs 4 & 5) or else run some additional numerical experiments that explicitly support these claims.
We very much appreciate this comment and have worked hard both to clarify our working definition of metastability as well as provide an additional numerical stability analysis. We feel that these additions greatly improve the paper and are thankful for the motivation to provide them. Specifically:

- **lines 222-235 (adaptive dynamics of radially symmetric carrying capacity and symmetric competition)**: Here we agree that there is a demonstrated lack of stability and clarify this in the paper. However, we also note that this case is likely a degenerate case based on the combined symmetry of the carrying capacity and competition functions. Given that this is the only case that does not settle to either a steady state or stable limit cycle, we feel that it's inclusion does not detract from the otherwise general comments on stability.
- **"reasonable biological timescales"**: We would first like to clarify that adaptive dynamics assumes infinitely small and frequent mutations which are what drive the smooth evolutionary trajectories through trait space. Adaptive dynamics is not on it's own able to model branching, so intermittently a "branching mutation" is attempted that creates a new mutant population nearby it's parent in order to initiate branching. So while the model is only running for 10^5 branching mutations, far more mutations are actually required to describe the dynamics. Additionally, while Zhao & Lieberman (2019) did find evidence of adaptive mutations in their very nice experiments, their estimation of $2 * 10^9 - 10^{12}$ mutations per microbiome per day is an estimation of SNP mutations, not mutations with phenotypic affect, or even more specifically mutations that affect metabolism, which is really what is being

modeled here. The number of these mutations is likely many orders of magnitude lower. That all being said, we very much appreciate the abstract nature of our model and feel that any parameterization attempts like this are more likely to give a false impression of the biological realism of our model than help convince the readers of the models import. We therefore simply removed the offending line.

- As for the overarching comment on the fuzzy notions of stability, we added a working definition of metastability to the paper (beginning on line 174 in the revision). Calculating true evolutionary stability was not analytically tractable, so instead we talk about metastability operationally as any system that resides in a particular state for longer than it took to converge to that state. In our case it often takes $\approx 5 - 50$ time units for a simulation to reach a limit cycle, which then continues for the the remaining $\approx 950 - 995$ time units (Please see Figure 3 for an example of convergence to the limit cycle in ≈ 20 time units or any of the complete 1000 unit trajectories in Figure 11 and included on-line `https://www.zoology.ubc.ca/~rubin/AltEvoDiversity/AD/`). So these systems are remaining in the limit cycle for at least an order of magnitude longer than it took to converge to them.

(2) There is some relevant theoretical literature on the evolution of stable, non-saturating ecosystems that the authors should discuss: Shoresh et al (PNAS, 2008) and Good et al (PNAS, 2019). In particular, the latter study obtained analytical solutions for the evolutionary stable states in a simple resource competition model with large numbers of substitutable resources, demonstrating the existence of a similar Red Queen like state ("diversification-selection balance") that does not saturate the available niche space. Given the apparent similarities with the present findings, the similarities and differences between these studies should be discussed more explicitly in the Introduction and Discussion sections.

- Thank you for pointing out this line of work. We have added a short discussion of these two papers in the discussion and think they add support to our conclusions of the commonality of unsaturated ecosystems (lines 444-450 in the revision). However, neither paper has stable unsaturated states nor red queen dynamics. Shoresh et al is more akin to evolutionary suicide continually opening up niche space and Good et al is an example of fast directional selection in one dimension limiting diversification in another dimension a la Ito and Dieckmann (2012, Evolutionary Ecology Research). Regardless, both studies are interesting and make positive contributions to the discussion.

(3) In Fig. 3, it would be useful to indicate what the initial population diversity was in each panel, as well as some rationale for how these particular examples were chosen (i.e. at random? to illustrate diversity of behaviors? etc). How are we supposed to interpret apparent similarities (or slight differences) between different panels?

- The figures felt a little too cluttered after we added the initial diversity as well, confusing an already fairly complicated figure. However, we agree that this is potentially interesting information for the readers, so we have included versions of the same figures with the initial diversity also displayed in the on-line supplement.

- We have clarified in the figure caption that each pane only differs based on the randomly generated initial community and that each pane represents an alternative level of stable diversity for the given parameters.

(4) For the cases in Figs. 3 & 4 where the "final evolutionary equilibrium" contains a single strain, would it be possible to analytical verify the stability claim? A few simple examples like this could go a long way toward addressing comment (1) above.

- Given the complexity of the dynamics, we were unable to analytically determine stability. This is a particularly tricky problem as the cyclic dynamics mean that neither areas of

positive invasion fitness nor the shape of the selection gradient can be used to determine whether a given mutant can invade, coexist with the resident population, and diversify in order to shift the community to a new state.

- In order to reinforce our claims of metastability, we added a numerical stability analysis for each of the simulations from Figure 4. This analysis is now included as Appendix 2. We extensively sample mutants around each resident that is alive at the end of the simulation (250 mutants per resident). We then restart the adaptive dynamics for 50 time steps (5% of the length of the original simulations) without allowing anymore mutations. Doing this we show that with small mutations, not a single mutant tested was able to invade, coexist with, and differentiate from the resident population. While we can never prove stability using this method, we feel that this is at least a very compelling argument for stability and a definitive proof of metastability.

(5) Much of the descriptive feel of the paper (for me at least) arose from the combinatorics of having 2 different carrying capacity models (quartic and radially symmetric) x 2 different types of interactions (symmetric vs asymmetric) x 3 different evolutionary dynamics (adaptive dynamics, individual-based, and PDE). In many cases, it wasn't clear when these models were simply meant to illustrate a diversity of possible behaviors vs proving a more targeted theoretical point.
My suggestion would be to pick one of the scenarios to describe in detail in the main text, and relegate the descriptive aspects of the others to an appendix whenever possible. The reclaimed space could then be used to specifically highlight how these other scenarios support or refute conclusions based on the "main" model alone.

- We have moved the PDE model to the supplement (now Appendix 3). We feel that the rest of the models are important to the general story. Adaptive dynamics is needed to computational ease, while the individual-based model highlights some interesting new dynamics that are a function of the demographic stochasticity and finite population size. The quartic and radially symmetric carrying capacities are perhaps slightly redundant, but we feel that it is important to show that these trends are at least partially separate from the functional forms. Given the already noted abstract nature of the model, we feel that it is important to show that this story is not a function of the specific model chosen, but at least present in the two cases investigated and thus likely a more global, and biologically relevant, story.

(6) While I agree that the PDE model can be regarded as an infinite population limit of the evolutionary dynamics, a large body of work in simpler systems (e.g. Hallatschek PNAS 2011) has shown that this is often a singular limit in scenarios where populations are continually adapting through new mutations. Thus, it is not necessarily clear that the PDE model should be a good approximation in large but finite populations, as implied by the discussion on 467.

- The PDE model was moved to the supplement, so this comment is of lesser consequence. Regardless, we aren't sure how "continually adapting through new mutations" is any different than the adaptive dynamics where that is true as well. Moreover, you noted above how it has been estimated that a microbiome accumulates somewhere on the order of $10^9 - 10^{12}$ mutations per day and Lenski's long-term evolution experiment, *E.coli* have been continually adapting via new mutations for over 60,000 generations (Good et al. 2017, Nature).

As for the general comment that the paper felt too descriptive, we hope that this was at least partially addressed through our clearer discussion of metastability and numerical stability analysis. As the reviewer notes, our model is fairly simple (biologically, not mathematically). We do not dispute this, but note that Lotka-Volterra dynamics has been arguably the backbone of a majority of ecological and evolutionary theory advances over the last century. While we think it is a worthwhile goal in it's own-right to understand these dynamics, we feel that getting too specific or analytical can lead to results that are too narrowly focused on the model itself and not the more

general results that can be interpreted in terms of real biological systems. While it is definitely a fine balancing act, we felt writing the paper with a slightly more descriptive tone (as well as including multiple functional forms to show some generality of the results) will make the work more accessible for a broader audience and tell a more useful biological story than if we had spent more time focusing on the intricacies and statistics of the specific modeling decisions we made.

Reviewer #2

(1) While the description of *what* the authors do when building the models is clear, the reasons behind their choices are not always as clear. For example, what is the reason behind the choice of the functional forms used for the competition functions α and for the carrying capacities K in Eqs (2)-(6) (beyond the ease of computation)? In other words, why did the authors choose a Gaussian form for the competition function, and quartic functions for the carrying capacities? Are there (even very simple) practical examples where these functional forms could be applied? I think that the article would gain a lot in interest to potential reader if these aspects were discussed more in depth.

- The model we analyze here is far from novel, so we did not want to spend too much space explaining decisions that are well supported and discussed at length in the literature. We refer you to Roughgarden (1979, Chapters 21 and 24) and Doebeli (2011, Chapter 3) for books that dive deeply into the academic history of these functional forms, their derivation from consumer-resource dynamics, as well as their limitations and assumptions.
- We have also added a short explanation and a few citations to our paper to help explain the model and the functional forms used (lines 115-117 in the revision).
- In addition to naturally limiting the viable phenotype space, using a quartic carrying capacity function avoids the structural instability of a Gaussian carrying capacity and Gaussian competition kernel that results in infinite branching (please see Doebeli 2011 chapter 3 and Pigolotti et al. 2010, Theoretical Ecology).
- This explanation has also been added to the paper (lines 91-93 in the revision).

(2) Related to the previous point, why is there a particular reason why the phenotype space that the authors consider is limited to [-2,2] in both directions?

- We refer to lines 87-89 in the revised manuscript. We did not artificially limit phenotype space to [-2,2], but the fourth-order nature of the carrying capacity kernel means that populations cannot be competitive and survive beyond those intervals. Really the viable phenotype space is within about [-1.5,1.5] but we chose to plot out to 2 to be conservative and for clarity of the figures.

(3) The authors do a very good job at describing the results that they have found. There are however some parts where I think a more thorough interpretation of the results would be desirable. For example, in the paragraph "Symmetric competition" (from line 214), is there any way to understand why the authors are seeing what they see? For example, is there a way to understand why the final state in phenotype space is a 4x4 grid, and why the points in the grid are in those exact positions? Do these properties depend on the particular values of the parameters? And if so, how? Is there any "universal" (i.e., not depending on the parameter values) property of the system in this case?

- Added short discussion of the grid pattern and a citation to previous work that goes into this in more detail (lines 239-243 in the revision).

(4) The paragraphs "Ecological dynamics" and "Evolutionary dynamics" under "Models and methods" (from line 76) are a bit cryptic at first reading, because several aspects of the models are discussed without showing the equations and the formulas to which the text refers to. Furthermore, some parts that are actually useful to understand how the models work are relegated in the Supplementary Information. I therefore suggest the authors to move the description of the properties of the models from the Supplementary Information to this part of the paper. I think that this way the article would gain considerably in clarity, particularly at first reading.

- Equations for the carrying capacities (Equations 1 and 2) and the competition kernel (Equation 3) were added to the main text from the supplement. The figure of the carrying capacity functions was also moved to the main text (now Figure 1) from the supplement to aid the readers understanding of these functions.

(5) I have not understood the reason behind the choice of Eq (12) as the form for the death rate in the individual-based model. I think the authors should explain why they have chosen this form more in detail.

- A short explanation was added explaining the derivation of this equation (lines 570-575 in the revision). In short, it is taken directly from the Lotka-Volterra equation. The per capita growth rate in the Lotka-Volterra equation $\left(1 - \frac{\sum_j^M \alpha(\vec{z}_j,\vec{z}_i)N_j(t)}{K(\vec{z}_i)}\right)$ can be understood as birth rate (1) minus the death rate $(\sum_j^M \alpha(\vec{z}_j,\vec{z}_i)N_j(t)/K(\vec{z}_i))$. Thus, the death rate for the individual-based model is just this, adjusted to work with discrete individuals instead of population densities.

(6) I think that the paragraph "Individual-based model" (from line 166) would be more clear if the authors spent a few words explaining how the clustering of individuals is made (even if they cite a few articles on line 178)

- First, we clarified it in the text that this clustering is purely an accounting method (lines 202-203 in the revision), so that we can compare meaningful levels of diversity, and has no impact on the model itself. Second, please see the text lines 200-202 in the revision. This line does not describe the specific algorithm we used, but it is enough to recreate our method of clustering perfectly. We feel that any further discussion of the clustering algorithm isn't salient to the paper and will only distract the readers.

(7) On lines 77-78, the authors say that they use "classic logistic consumer resource ecological dynamics". This choice of words, and in particular "consumer resource", is potentially misleading since it could refer to MacArthur's consumer-resource framework for modelling population dynamics. I therefore invite the authors to say "classic logistic Lotka-Volterra ecological dynamics".

- Changed.

(8) There are some typos throughout the text (e.g., "stats" instead of "states" at the end of the Author Summary, "affect" instead of "effect" in line 92, "$\alpha(z_i, z_j) = \alpha(z_i, z_j)$" instead of "$\alpha(z_i, z_j) = \alpha(z_j, z_i)$" in line 96, etc.). I recommend the authors to check again the whole text for typos.

- We apologize for these typos and made the necessary corrections.

Reviewer #3

l.2-3: "how different patterns of biodiversity are maintained" or "how biodiversity is maintained"?

- Changed.

l.6-7: "'stable coexistence of diversity" or "stable coexistence of species"?

- Changed.

l.7: "For communities to coexist" or "For species to coexist"

- Changed.

l.9-10 This paragraph starts with talking about two ways of diversifying, but we never hear about "exploration". Why introduce it?

- We refocused the sentence to focus on niche packing, but left the mention of exploration in there as we think it is both interesting and gives historical context to the broader question of the evolution of diversity.

l.27: when you say "alternative ESS's", do you mean local evolutionary stability or global?

- Local. Clarified by changing "uninvadable by a mutant" to "univadable by a mutant of small effect" (lines 27-28 in the revision).

l.64: introduce the alternative explanation for large mutations (species invasion) already here

- Added (lines 65-66 in the revision).

l.98: The phrase "asymmetric competition" is usually taken to mean when a larger trait value is better – "Asymmetric competition arises when, during an encounter between two or more individuals for some limited resource, these resources are divided up unequally: the larger individual wins the contest, the territory holder keeps the territory, the taller plant gets more light." from ref [31]. That is not how it is used in this paper, which was a major point of confusion for me until I explored the competition function myself. This should be at least pointed out, but preferably a different phrase used.

- We actually think that quote perfectly summarizes the asymmetric competition function we use here. Asymmetric competition simply refers to when the competitive effect of one individual on another does not just depend on their distance apart in trait space, but also on their positions in trait space (e.g., one is taller). Or alternatively, the competitive effect of x on y does not necessarily equal the competitive effect of y on x, but also depends on the both x and y's phenotypes.

- As we explain in the paper (lines 107-110 in the revision), the asymmetric term used here in phenomenological and can be thought of as the first-order term in a Taylor expansion of some higher-order competition function.

model description: I think it would help to give the equation of the competition kernel in the main text, and to have a figure with it and the two carrying capacity functions

- All added (now Equations 1 and 2 and Figure 1).

There seems to be contradiction between l.122 "when a favorable mutation arises, it out-competes the resident" and l.134 talking about potential coexistence of mutant and resident. Maybe add "usually" to l.122?

- Deriving the canonical equation of adaptive dynamics does assume that all favorable mutations out compete their parent. Coexistence of a mutant and parent is only possible when a mutant population is manually split off from the parent in the algorithm described in the text. Please see our comment bellow explaining this confusion.

l.143 "adaptive dynamics" is often used to model branching/"speciation", so why say it can't be?

- Adaptive dynamics can only describe the movement of species in trait space. It can be used to investigate when branching may occur by looking at the curvature of the invasion fitness landscape, but the adaptive dynamics equations themselves do not explicitly model branching. To do this, the classical algorithm, and the one employed here, is to periodically split a population, then recalculate the adaptive dynamics with one additional population. If the mutant species diverges from it's parent, a branching event has occurred. If it dies out or fails to move away from the parent, the system is reverted back to the original state. The terminology surrounding this can be confusing because "Adaptive Dynamics" may be used to refer to the entire algorithm that includes these manual branching events or just to the canonical equation of adaptive dynamics which does not model branching.
- In order to help clarify what we are referring to, we change the text to say that the canonical equation of adaptive dynamics cannot describe branching.

l.146 "well described algorithm" – well described where?

- Citation added (citation #18).

l.206 not clear what "higher order" means here, or where it was shown that this implies branching. Got a reference?

- Higher order was just meant say that the function inside the exponent is to the fourth power for the carrying capacity function and the second for the competition function.
- We replaced this phrase by a more detailed explanation: "Because the carrying capacity functions we investigate here both have a broader maximum at the origin than that of the Gaussian competition kernel, the origin is always the minimum of the invasion fitness and there necessarily exists a branching point at the origin. Thus the possibility of evolutionary coexistence between two or more phenotypes is guaranteed."

l.310 define "metastable"

- Section defining metastability was added.

Fig. 4-5: what does "initial ecological equilibrium" and "final evolutionary equilibrium" on axes mean? initial & final species richness might be better

- Changed to proposed axes titles.

l.386 this isn't really a resource competition model, more phenomenological

- Changed from "resource competition" to "Lotka-Volterra competition"

l.419 It's a leap to suggest that most natural systems are unsaturated and undergoing Red Queen dynamics based on this model with MANY unrealistic assumptions (no immigration, Lotka-Volterra competition, etc.)

- Changed from "most systems" to "many competitive communities"

eqn (8): is that ∂f or $\partial \alpha$?

- Thank you for catching the error. Changed.

l.526 it's well known that the relative evolutionary rates can affect the stability of evolutionary equilibria. Am I missing something?

- We agree that nontrivial versions of the mutational variance-covariance matrix can indeed affect evolutionary dynamics. However, adding any off-diagonal elements to the M matrix is always mathematically equivalent to a diagonal M matrix with different asymmetric competition coefficients. As we generate competition coefficients randomly, the asymmetric dynamics described here are sufficient to describe the full range of possible dynamics without the necessary complications of a nontrivial mutation variance-covariance matrix.

eqns (10) & (11): how do these follow from (7) and (8)? seems like there are many terms missing

- The equations in the text are correct (they are now equations 13 and 14). Because the partial derivative of the competition kernel is evaluated at $y = x$, the Guassian part of the competition kernel disappears. That leaves us with only the partial derivative of the asymmetric component with respect to the mutant evaluated at $y = x$, which equals $\sum_{l=1}^{d} b_{kl} x_l$. For the second term, the partial derivative of the carrying capacity with respect to the parent is divided by the carrying capacity itself. Because the carrying capacity is an exponent, only the derivative of the inner function remains. i.e., for the quartic case, $\frac{\partial K(\vec{x})}{\partial x_i} \frac{1}{K(\vec{x}_i)} = \exp\left(-\sum_{k=1}^{d} \frac{\vec{x}_k^4}{4}\right)\left(-\vec{x}_i^3\right) \frac{1}{\exp\left(-\sum_{k=1}^{d} \frac{\vec{x}_k^4}{4}\right)} = -\vec{x}_i^3$. Putting these together, we are left with equation 10.

- This explanation was added (lines 542-549 in the revision).

When I attempted to replicate the results (quartic fitness function), I found that periodically invading the co-evolutionary limit cycles could indeed build up to the final ESS. What did I do differently? When I introduced new species, I did not first check that they had positive invasion fitness, but instead just followed their own trait (and population) dynamics. They easily diverged from each other, and never reached low population densities along the way either. After 7 species, the limit cycling was replaced by an evolutionary equilibrium, which ended up with 9 species in a skewed grid. So, perhaps a hidden assumption required to find the authors' metastable cycles is the strict separation of ecological and evolutionary time scales. Worth looking into!

- We are very happy that you attempted to replicate our results, but without the algorithm you used and knowing your parameterization, it is hard for us to comment on how your results differ from the ones we present here. Regardless, we are not really sure how checking for positive invasion fitness would be able to change the dynamics. Checking this is strictly a computational efficiency step. As mutants are introduced at low population size, any mutant introduced with negative invasion fitness will have a negative growth rate and immediately go extinct. The code we use to generate these results is included on-line and we welcome any further discussion of our algorithm and results.

- Also, we very much agree that investigation into how relaxing the separation of timescales and the resulting eco-evolutionary dynamics affects the maintenance of diversity and niche packing. However, that is likely at least another paper in it's own right, and too much to include here.

In addition to the revision made based on the comments from the reviewers, we fixed a few small typos and made a couple minor adjustments to the text.
