# Supplementary material for: Evolution to alternative levels of stable diversity leaves areas of niche space unexplored
Source: PLoS Comput Biol. 2021 Jul 28;17(7):e1008650. doi: 10.1371/journal.pcbi.1008650 (PMC8351994; doi:10.1371/journal.pcbi.1008650)
Supplement: Attachment — Submitted filename: response_to_reviewers_v2.pdf [file pcbi.1008650.s005.pdf]

Response to Reviewer Comments

Reviewer #1

I'd like to thank the authors for their work in revising the manuscript. Most of my comments have been addressed, except for one remaining point on the PDE model. I agree with the authors that this is not of central importance to the revised manuscript, but for the sake of technical accuracy in the Appendix, I think it is still important to address.
I had a little trouble following the logic in the authors' response, so I'll try to rephrase: I'm not trying to argue that the large N limit is unrealistic or not worth looking at, but rather that as a technical matter, the N=infinity PDE model is *NOT* necessarily the same as the N→infinity limit of the individual-based model. I.e., in an asymptotic sense, the leading order contribution as N→infinity is not necessarily the same as setting N=infinity from the start. This is known to be the case in the simple traveling wave models studied by Hallatschek and others, and I don't see why we'd expect it to be any different for the more complicated models studied here.
With that in mind, my only issue w/ the phrasing in the revised manuscript is line 700: "The large population limit of the individual based model gives the deterministic formulation of partial differential equations." A simple fix would be to change the beginning to "the infinite population version" or "the deterministic limit" instead.

- Changed to "the infinite population version" as suggested (the first line of the second paragraph of S3 Appendix in the Supplementary Information).

Reviewer #3

Reviewer #3 from the previous round here. This remains an interesting study on the existence of local ESS's and Red Queen evolutionary limit cycles in eco-evolutionary models, which I'll be glad to see in print.
That said, some of my comments from the previous version were not accepted, which I think will lead to some confusion for some readers. Particularly, the authors take "adaptive dynamics" to mean only directional selection of extant species, not the initiation of new lineages through evolutionary branching. This deviates from the commonly accepted usage starting with the foundational papers of adaptive dynamics (Metz et al. 1996, Geritz et al. 1998). It leads to contradictory statements like "when a favorable mutation arises, it out-competes the resident" (l.130) and "when mutual invasibility occurs ... the two phenotypes can coexist indefinitely" (l.140-142) and "unlike adaptive dynamics, evolutionary branching is an emergent property" (l.210). The fix is simple: add "usually" to l. 130 and don't use "adaptive dynamics" as a synonym for "the canonical equation of adaptive dynamics" (e.g. l.155, 164, and throughout).

- As suggested, "usually" added to line 130 and "canonical equation" added in three places (lines 145, 156, and 166 in the new manuscript) to clarify this distinction.

l.72 The conclusion that "locally stable, low levels of diversity are likely ubiquitous in nature" is a bit strong. I think the authors have show that it is possible, but it's certainly not the only outcome in the authors' models, which also rely on particular assumptions.

- Changed "ubiquitous" to "present."

l.104 reverse the subscripts in the second part

- Fixed. Thank you for catching the error.

l.150 The canonical equation isn't described above.

- Changed “described” to “introduced” and pointed the reader toward Appendix S1 with the derivation of the adaptive dynamics.

l.234 fix
alpha

- Fixed.

Fig. 2 Put the range of the axes on the axes, not in the figure legend.

- The confusing line was removed from the caption. The figures already display the range.

l.246 For clarity, I’d say “almost flat and nearly equal to zero”

- Changed.

throughout - suppress unintentional new paragraphs after equations with (e.g. l.87, 111, ...)

- The new paragraphs we suppressed after lines 87 and 111 as requested. Other equations already had new paragraphs suppressed.

In addition to the revision made based on the comments from the reviewers, we fixed a few small typos and made a couple minor adjustments to the text.
